# Genome Sequence of *Bacillus thuringiensis* Strain Btm27, an Egyptian Isolate Highly Toxic to Cotton Leafworm

**DOI:** 10.1128/genomeA.00446-15

**Published:** 2015-05-14

**Authors:** Brigida Rusconi, Yue Chen, Sara S. K. Koenig, Ehab R. El-Helow, Mark Eppinger

**Affiliations:** a University of Texas at San Antonio, Department of Biology and South Texas Center for Emerging Infectious Diseases, San Antonio, Texas, USA; b University of Alexandria, Department of Botany and Microbiology, Alexandria, Egypt; c Pharos University in Alexandria, Department of Microbiology and Immunology, Alexandria, Egypt

## Abstract

*Bacillus thuringiensis* is a potent microbial control agent against insect pests. Here, we present the draft genome of the Egyptian strain Btm27 that shows high toxicity toward the cotton leafworm. The genome contains three insecticidal genes *cry1Ac9*, *cry2Ab1*, and *vip3V* that have been implicated in conferring toxicity toward lepidoptera.

## GENOME ANNOUNCEMENT

*Bacillus thuringiensis* has been successfully used as a biopesticide to control many agricultural pests and insect vectors of human disease (1). The entomopathogenicity of *B. thuringiensis* is attributed to the expression of a broad variety of species-specific toxic proteins, driven by evolutionary sequence divergence and recombination events (2). These include largely plasmid-borne (3) vegetative insecticidal proteins (Vip), sporulation associated crystal proteins (Cry), and cytolytic toxins (Cyt) (4). Besides its role as biopesticide, further promising biotechnological applications included the production of industrially important enzymes (5) for cytotoxic effects on cancer cells (6). As recently demonstrated by Alfazairy et al. (7), the sequenced Egyptian strain Btm27 is a potent control agent against cotton leafworm. Structural and functional genomic analyses of the strain will allow one to better characterize its insecticidal efficiency and biotechnological potential.

Total genomic DNA was extracted with QIAamp DNA minikit according to the manufacturer’s protocol. Sequencing was performed on the Illumina MiSeq platform using a paired-end library with 300-bp read length. The draft genome was assembled with Spades 3.0 (8). The average G+C content of 35% and total length of 5,871,441 bp of the obtained Btm27 sequences are in accordance with the findings for other *B. thuringiensis* genomes (9, 10). All contigs were annotated using the PROKKA annotation pipeline (11) and a total of 5,050 coding sequences, 79 tRNAs, 11 rRNA operons, and four circular plasmids were identified. A BLASTn (9) analysis of the Btm27 contigs against the NCBI nonredundant (nr) database identified *B. thuringiensis* serovar *kurstaki* strain YBT-1520 as the closest relative. Draft sequences were further compared at the nucleotide and protein levels against a *B. thuringiensis* specific plasmid database on the Galaxy platform (10). The results suggest that the Btm27genome is organized into five replicons: a circular chromosome and four plasmids that show high similarity to plasmids pBMB293, pBMB8513, and pBMB400 in *B. thuringiensis* subspecies *kurstaki* strain YBT-1520, and pBMB65 in *B. thuringiensis* subspecies *kurstaki* strain HD-1. Utilizing BtToxinScanner, we identified three toxin genes in the Btm27 genome and classified them as *cry1Ac9*, *cry2Ab1*, and *vip* (12). The predicted *vip* coding sequence was further compared to curated Vip proteins in Uniprot (13) and showed 100% identity to Vip3V, which has toxic activity against lepidopteran larvae (14). Interestingly, all three toxins have been demonstrated to be highly active against a range of lepidopteran insect pests (15, 16). The limited variety of insecticidal genes carried by Btm27 suggests that the strain is an ideal candidate for specified pest control preventing unwanted toxic effects on taxonomically unrelated insects. Genome annotation also revealed the presence of genes responsible for the expression of biotechnologically important degradative enzymes, such as chitinases and proteases that cover serine protease, neutral protease, and metalloprotease activities. The availability of the genome sequence of *B. thuringiensis* strain Btm27 lays the foundation for further structural and functional analyses to fully elucidate its biotechnological potential.

### Nucleotide sequence accession number. 

This genome sequence has been deposited in GenBank under the accession no. JWJY00000000. Strain Btm27 has been deposited into the Bacillus Genetic Stock Center (BGSC) collection as 4AC2.
